# *GPX3* Variant Genotype Affects the Risk of Developing Severe Forms of COVID-19

**DOI:** 10.3390/ijms242216151

**Published:** 2023-11-10

**Authors:** Marko Markovic, Jovan Ranin, Zoran Bukumiric, Djurdja Jerotic, Ana Savic-Radojevic, Marija Pljesa-Ercegovac, Tatjana Djukic, Marko Ercegovac, Milika Asanin, Ivana Milosevic, Goran Stevanovic, Tatjana Simic, Vesna Coric, Marija Matic

**Affiliations:** 1 Clinic of Infectious and Tropical Diseases, University Clinical Centre of Serbia, 11000 Belgrade, Serbia; markovicdrmarko@gmail.com (M.M.); njranin@gmail.com (J.R.); ivana.milosevic00@gmail.com (I.M.); goran_drste@yahoo.com (G.S.); 2 Faculty of Medicine, University of Belgrade, 11000 Belgrade, Serbia; zoran.bukumiric@med.bg.ac.rs (Z.B.); djurdja1.jovanovic@gmail.com (D.J.); ana.savic-radojevic@med.bg.ac.rs (A.S.-R.); m.pljesa.ercegovac@gmail.com (M.P.-E.); tatjana.djukic@med.bg.ac.rs (T.D.); ercegovacmarko@gmail.com (M.E.); masanin@gmail.com (M.A.); tatjana.simic@med.bg.ac.rs (T.S.); 3 Institute of Medical Statistics and Informatics, 11000 Belgrade, Serbia; 4 Institute of Medical and Clinical Biochemistry, 11000 Belgrade, Serbia; 5 Centre for Excellence for Redox Medicine, Pasterova 2, 11000 Belgrade, Serbia; 6 Clinic of Neurology, University Clinical Centre of Serbia, 11000 Belgrade, Serbia; 7 Clinic of Cardiology, University Clinical Centre of Serbia, 11000 Belgrade, Serbia; 8 Department of Medical Sciences, Serbian Academy of Sciences and Arts, 11000 Belgrade, Serbia

**Keywords:** oxidative stress, COVID-19, disease progression, antioxidant genetic profile, GPX3, inflammatory markers

## Abstract

In SARS-CoV-2 infection, excessive activation of the immune system intensively increases reactive oxygen species levels, causing harmful hyperinflammatory and oxidative state cumulative effects which may contribute to COVID-19 severity. Therefore, we assumed that antioxidant genetic profile, independently and complemented with laboratory markers, modulates COVID-19 severity. The study included 265 COVID-19 patients. Polymorphism of G*STM1*, *GSTT1*, *Nrf2 rs6721961*, *GSTM3 rs1332018*, *GPX3 rs8177412, GSTP1 rs1695*, *GSTO1 rs4925*, *GSTO2 rs156697*, *SOD2 rs4880* and *GPX1 rs1050450* genes was determined with appropriate PCR-based methods. Inflammation (interleukin-6, CRP, fibrinogen, ferritin) and organ damage (urea, creatinine, transaminases and LDH) markers, complete blood count and coagulation status (d-dimer, fibrinogen) were measured. We found significant association for COVID-19 progression for patients with lymphocytes below 1.0 × 10^9^/L (OR = 2.97, *p* = 0.002). Increased IL-6 and CRP were also associated with disease progression (OR = 8.52, *p* = 0.001, and OR = 10.97, *p* < 0.001, respectively), as well as elevated plasma AST and LDH (OR = 2.25, *p* = 0.021, and OR = 4.76, *p* < 0.001, respectively). Of all the examined polymorphisms, we found significant association with the risk of developing severe forms of COVID-19 for *GPX3 rs8177412* variant genotype (OR = 2.42, *p* = 0.032). This finding could be of particular importance in the future, complementing other diagnostic tools for prediction of COVID-19 disease course.

## 1. Introduction

Clinical course and manifestations of severe acute respiratory syndrome coronavirus 2 (SARS-CoV-2) infection and the disease caused by this virus (COVID-19) may vary, from asymptomatic to severe [1,2,3]. A particular challenge in COVID-19 treatment is to recognize patients who are at increased risk for developing a severe form of the disease in its early stage [4,5]. To date, several risk factors for the disease progression have been identified, such as arterial hypertension, diabetes mellitus, renal impairment, certain malignancies, older age and male sex [4,6,7,8]. However, a certain number of patients without any of these known risk factors develop a severe form of COVID-19. According to the latest reports, the overall mortality from COVID-19 is 3.77–5.4%, but it increases to 41.1–61.5% in severely or critically ill patients [9]. Biochemical markers important for monitoring and prognosis of COVID-19 patients are inflammation parameters (interleukin-6 (IL-6), C-reactive protein (CRP), fibrinogen, ferritin), leucocyte count, platelets, biochemical markers of tissue damage (urea, creatinine (Cr), transaminases (AST, ALT), lactate-dehydrogenase (LDH)), as well as coagulation parameters (fibrinogen and d-dimer) [10,11,12]. These markers may be a helpful tool in identifying patients with an increased risk of developing a more severe form of COVID-19 [4,13]. 

Previous studies indicated that oxidative stress plays one of the key roles in the development of severe acute respiratory failure caused by SARS-CoV-2 infection [14,15]. It is possible that SARS-CoV-2, like other RNA viruses, can induce oxidative stress [14,16,17]. It is assumed that oxidative stress in COVID-19 infection has multiple roles in various processes, such as the interaction of the virus with host cells and replication of the virus, in addition to the involvement in an enhanced inflammatory response as well as in damage of various types of tissues and organs [18,19]. Moreover, the hyperproduction of free radicals, among which reactive oxygen species (ROS) are the most important, as well as the inability of antioxidant mechanisms to compensate them further contribute to the development of more severe clinical manifestations of the disease [20,21]. Moreover, SARS-CoV-2 infection activates phagocytic cells, which leads to excessive production of ROS, while antioxidant protection mechanisms are insufficiently expressed.

In order to protect biologically important macromolecules against oxidative damage, numerous non-enzymatic and enzymatic antioxidant systems exist in the human body [22]. The immediate line of enzymatic antioxidant protection consists of superoxide dismutase (SOD) isoenzymes, while the first line of defense against ROS includes glutathione peroxidase (GPX) and glutathione transferase (GST) isoenzymes [23]. On the other hand, one of the most important regulatory antioxidant proteins is Nrf2 (nuclear factor-erythroid-2-related factor 2), a transcription factor that regulates basal activity and coordinated gene expression for the aforementioned antioxidant enzymes, especially certain classes of cytosolic GSTs, such as GSTA-1, GSTM-1, GSTM-3 and GSTP-1 [24,25]. Several studies have shown decreased expression of Nrf2 in patients with COVID-19 [26,27]. The activity of antioxidant enzymes and regulatory proteins is also influenced by genetic heterogeneity due to the presence of deletion polymorphisms (GSTM1 *null* and GSTT1 *null* variant genotypes) as well as single-nucleotide polymorphisms (SNPs) in both coding regions (GSTP1rs1695*Ile/Val + Val + Val, GSTO1rs4925*Ala/Asp + Asp/Asp, GSTO2 rs156697*Asn/Asp + AspAsp, SOD2rs4880*Ala/Val + Val/Val, GPX1rs1050450*Pro/Leu + Leu/Leu variant genotypes) and non-coding gene regions (Nrf2rs6721961*CA + AA, GSTM3rs1332018*AC + CC, *GPX3*rs8177412*TC + CC variant genotypes) [28,29,30,31]. Many of these variations found within genes encoding for leading regulatory and catalytic antioxidant proteins make a direct impact on the protein structure, function and expression, reshaping their activity and substrate diversity as well.

Oxidative stress seems to impose a certain milieu in COVID-19 pathogenesis. Previous studies have pointed out that the genetic profile of antioxidant enzymes plays a very important role in risk assessment and prognosis of disease course in patients with COVID-19 [32]. Our previous results have shown that SNPs found in genes encoding for antioxidant enzymes, such as GSTP1, GSTM3, GSTO1 and GSTO2, were significantly associated with the probability of getting COVID-19 along with developing certain clinical manifestations [29,33]. Moreover, it has been indicated that individuals with the GSTT1 *null* genotype have a positive correlation with the mortality from COVID-19 [34]. With the intention to further expand the knowledge on the interplay between inflammation and oxidative stress in SARS-CoV-2 infection course, in this study we aimed to analyze the association of genetic polymorphisms of regulatory and catalytic antioxidant proteins with the severity of COVID-19 disease, both independently and complemented with commonly assessed laboratory parameters.

## 2. Results

The distribution of patients according to the severity of COVID-19 is presented in Table 1. Most of our study population had moderate disease (53.2%), severe and critical form was present in 25.7% of patients, while 21.1% had mild clinical presentation. As presented, our study group consisted predominantly of males (61%), and the mean age was 56 years. The most frequently observed comorbidity was hypertension, which was present in half of our patients. Other comorbidities, such as diabetes mellitus and obesity were also present among the studied population in 13% and 32% of patients, respectively. More than half of our study participants declared themselves to be non-smokers (55%).

Results of selected laboratory parameters of inflammation, complete blood count, biochemical parameters of damage to certain organs and indicators of coagulation status in patients with COVID-19 at the time of hospital admission are shown in Table 2.

Among blood count parameters, leucopoenia was present in 22%, and lymphopenia was present in 32% of study participants. Also, thrombocytopenia was present in 22% of our study participants. As expected, we observed significant number of participants with elevated inflammatory markers such as IL-6 (51%), CRP (73%) and ferritin (32%) on admission. When it comes to coagulation parameters, 50% of our participants had elevated d-dimer, while 37% had elevated fibrinogen at the time of admission. LDH and AST activities were among markers of organ damage that were also high on admission in a significant number of patients, in 48% and 34% of participants, respectively. 

Using ordinal repression, we analyzed the association between specific laboratory parameters and the risk of developing a severe form of COVID-19 (either medium or severe form) as shown on Table 3. Among blood count parameters, statistically significant association for progression of COVID-19 was found for patients with lymphocytes below 1.0 × 10^9^/L (OR = 2.97, *p* = 0.002). Elevated concentration of inflammatory markers IL-6 and CRP was also associated with progression of the disease (OR = 8.52, *p* = 0.001, and OR = 10.97, *p* < 0.001, respectively). Overall, patients with low lymphocyte count, elevated IL-6 and CRP were at 3 to 11 times increased risk of progressing to a more severe form of COVID-19. We also found that elevated fibrinogen concentration carries increased the risk of disease progression (OR = 2.29, *p* = 0.029). In addition, patients with elevated AST and LDH activities were susceptible to a more severe COVID-19 form (OR = 2.25, *p* = 0.021, and OR = 4.76, *p* < 0.001, respectively). Namely, our results have indicated that patients with elevated AST and LDH activities were at approximately 2.3 and 5 times greater risk (*p* = 0.021 and *p* < 0.001) of developing more severe COVID-19, respectively. We found no association for creatinine and urea concentrations. 

Among analyzed gene polymorphisms, we found no significant association for null/variant genotypes of *GSTM1* (OR = 0.87, *p* = 0.673), *GSTT1* (OR = 0.75, *p* = 0.455), *Nrf2rs6721961* (OR = 0.8, *p* = 0.538), *GSTM3rs1332018* (OR = 1.76, *p* = 0.091), *GPX3rs8177412* (OR = 1.51, *p* = 0.247), *GSTP1rs1695* (OR = 1.02, *p* = 0.978), *GSTO1rs4925* (OR = 1.23, *p* = 0.551), *GSTO2rs156697* (OR = 1.23, *p* = 0.551), *SOD2rs4880* (OR = 0.65, *p* = 0.287) and *GPX1rs1050450* (OR = 1.02, *p* = 0.952) or the risk for the disease progression when ordinal regression was adjusted to gender, age, selected comorbidities and smoking status (Table 4). 

The associated effect of laboratory parameters and gene polymorphisms for proteins important in the regulation of redox homeostasis with the risk of developing a more severe form of COVID-19 is presented in Table 5. In our study, we found no significant association for the null/variant genotypes related to the following gene polymorphisms: *GSTM1*, *GSTT1, Nrf2 rs6721961*, *GSTM3rs1332018*, *GSTP1rs1695*, *GSTO1rs4925*, *GSTO2rs156697*, *SOD2 rs4880* and *GPX1 rs1050450* (*p* > 0.05). However, the carriers of *GPX3* variant genotype (GPX3*TC + CC), associated with decreased intracellular enzyme expression, were at 2.42-fold increased risk for developing more severe forms of COVID-19 compared to the carriers of the *GPX3* genotype of the referent genotype (OR = 2.42, *p* = 0.032) when the OR adjusted to inflammatory markers (leukocyte, lymphocyte and platelet count, IL-6, CRP, fibrinogen, ferritin, d-dimmer concentration) was computed in ordinal regression. 

## 3. Discussion

Since oxidative stress has an important role in and SARS-CoV-2 susceptibility and infection, we assumed that variations in catalytic and regulatory antioxidant proteins, independently and complemented with commonly assessed laboratory parameters, may also modulate COVID-19 severity. In our study, we found that inflammatory markers such as lymphocyte count, CRP and IL-6 levels correlated with the risk for the disease progression. In addition, laboratory markers of organ damage, such as AST and LDH, are also shown to be of prognostic value for development of more severe forms of COVID-19. On the other hand, we did not find significant association for the null/variant genotypes of proteins important in redox homeostasis regulation (*GSTM1*, *GSTT1*, *Nrf2 rs6721961*, *GSTM3rs1332018*, *GSTP1rs1695*, *GSTO1rs4925*, *GSTO2rs156697*, *SOD2 rs4880 and GPX1 rs1050450)* with the risk of developing a more severe form of COVID-19. However, we found that carriers of the *GPX3 rs8177412* variant allele were at approximately 2.5-fold increased risk for developing more severe forms of COVID-19 compared to the carriers of the *GPX3* referent genotype when adjusted to inflammatory markers.

Several laboratory parameters have been proposed to correlate with a poorer outcome of COVID-19 [10,13,35]. Our study reported that low lymphocytes and elevated levels of CRP and IL-6 on admission correlate with the risk of progression to a more severe form of COVID-19, which is similar to results of numerous studies. Namely, the meta-analysis of Henry BM. et al., which included about 3400 patients and 33 laboratory parameters, shows that patients with severe and fatal disease had significantly increased white blood cell (WBC) count and decreased lymphocyte and platelet counts compared to patients with mild disease [11,36]. Another meta-analysis of Huang I. et al., with total of 5350 patients, showed that an elevated serum CRP, PCT, D-dimer and ferritin were associated with a poor outcome in COVID-19 [37]. Worsening of laboratory markers, including a decrease in the number of lymphocytes, elevated neutrophil/lymphocyte ratio (NLR), CRP, ferritin, TNF, IL-6, IL-10, d-dimer and PT, was registered during the progression of the disease [19,38,39]. It is well established that TNF and IL-6 trigger CRP synthesis in the liver, while IL-8 increases neutrophil recruitment [40,41]. IL-6 activates neutrophils and monocytes, which leads to increased production of ROS [42]. At the same time, ROS can elevate levels of IL-6 in this vicious circle of cytokine and oxidative stress storm [43]. That is one of the reasons why high levels of IL-6 during COVID-19 are related to high mortality rates in patients treated in the ICU [44]. Our study also found that high levels of organ damage laboratory markers, such as AST and LDH, are associated with more severe forms of COVID-19. Similar results were reported by Battaglini D. et al., who have shown that on admission, lower lymphocyte and platelet counts and higher values of ferritin, D-dimer, LDH and AST correlated well with clinical severity of COVID-19 patients [45]. Moreover, a large meta-analysis with a total of 15,354 COVID-19 cases showed that cardiac biochemical markers, including LDH, are associated with the severity of COVID-19 [45]. 

Non-communicable diseases, such as arterial hypertension and diabetes mellitus, that are seen more frequently in the elderly population are associated with increased formation of reactive oxygen species. Moreover, increased BMI may be regarded as state of chronic systemic inflammation with persistently elevated levels of pro-inflammatory cytokines (such as IL-6 and consequently CRP) that is perplexed with disturbed redox homeostasis. Presumably, in patients with the aforementioned conditions, extensive exposure to free radicals produced by cigarette smoking may further contribute to the crosstalk between inflammation and oxidative stress. Therefore, aged, obese, diabetic and hypertensive smokers may represent the frailest group of patients in whom SARS-CoV-2 infection can further amplify the vicious cycle of oxidative stress and inflammation that mutually enforce each other. Such patients would highly benefit from the assessment of non-specific inflammatory parameters on admission, usually procured from routine laboratory practice. Still, the aforementioned conditions (hypertension, diabetes mellitus, obesity and smoking) are rather frequent in general population. However, only a certain number of affected individuals will eventually develop severe forms of COVID-19. This can be partially explained by variations found in genes encoding proteins for enzymatic antioxidative defense.

Our previous results of association of antioxidant genetic profile and COVID-19 susceptibility have shown that SNPs found in genes encoding GSTP1, GSTM3, GSTO1 and GSTO2 were significantly associated with disease susceptibility [29,33]. Namely, Coric V. et al. have shown that individuals carrying the GSTP1* (Ile105Val rs1695) or GSTP1* (Ala114Val rs1138272) as well as GSTM3*AC (rs1332018) variants were less prone to developing COVID-19, while Djukic T. et al. reported that individuals carrying GSTO1*AA and GSTO2*GG polymorphic variants had a significantly increased COVID-19 risk. However, regarding developing a more severe form of COVID-19, in this study we found no significant association for the null/variant genotypes of the following gene polymorphisms: *GSTM1*, *GSTT1*, *Nrf2 rs6721961*, *GSTM3rs1332018*, *GSTP1rs1695*, *GSTO1rs4925*, *GSTO2rs156697*, *SOD2 rs4880* and *GPX1 rs1050450.* Our results are in contrast with a study of Abbas M. et al. conducted on a population of North American natives, which reported that the frequency of null alleles *GSTM1−/−* and *GSTT1−/−* were more prevalent in patients with severe COVID-19 compared to those who had a milder clinical presentation [46]. In another study, Saadat M. et al. [34] suggested that individuals carrying *GSTT1−/−* genotype had a higher COVID-19 mortality rate. A recent Polish study conducted by Orlewska K. et al. reported that *GSTP1 Ile/Val* genotype was associated with higher risk of developing a severe form of the disease. However, they found that *GSTM1 null* and *GSTT1 null* genotypes had no significant association with risk of developing severe forms of COVID-19, which is in agreement with results of our study [47]. 

When we adjusted analysis to inflammatory markers, we found that among all examined polymorphisms only the *GPX3* variant allele was associated with more severe forms of the disease. Namely, carriers of *GPX3 rs8177412* variant allele were at approximately 2.5-fold increased risk for developing more severe forms of COVID-19 compared to the carriers of the *GPX3* referent genotype. Results of our previous study have shown that the *GPX3 rs8177412* variant allele is also associated with COVID-19 susceptibility [28]. Among the eight recognized GPX isoforms, GPX3 is the only one localized within the extracellular space. Present within plasma, it serves as a major antioxidant enzyme responsible for scavenging hydrogen peroxide and organic hydroperoxides [48,49]. The occurrence of a polymorphism within the *GPX3* gene, denoted as *rs8177412*, leads to downregulation of gene transcription and consequently to distinct reduction in the GPX3 plasma activity [30,50]. This reduction in plasma GPX3 activity subsequently contributes to increased extracellular oxidative stress, diminished bioavailability of nitric oxide and the stimulation of platelet activation. These activated pathways of oxidative stress exert a negative impact upon the vascular endothelium and trigger inflammatory processes contributing to the pathophysiology of COVID-19 and potentially to the progression of more severe forms of the disease [21,51,52].

Finally, some limitations related to this study should be addressed. Apart from the relatively small sample size, selected oxidative-stress-related genetic variants were assessed without taking into account the effect of other ones. Moreover, the study comprised virus variants present during the second wave of the epidemic in Serbia, as reported by Miljanovic et al. [53]. Therefore, the potential impact of specific mutations on the severity of symptoms was not considered. Another potential drawback of this study comprises the lack of knowledge regarding patients’ recovery time and its association with assessed gene variants. Finally, other confounding factors might have posed a certain effect, although regression analysis was used to minimize the impact of non-genetic factors on the results of our study.

## 4. Materials and Methods

The study included 265 patients who were consecutively admitted to hospital treatment at the Infectious and Tropical Diseases Clinic of University Clinical Center of Serbia, between September 2020 and June 2021. The criteria for including patients in the study were SARS-CoV-2 infection, confirmed by a positive PCR test in nasopharyngeal swab samples, between June 2020 and October 2020. The excluding criteria were the presence of an associated infection (human immunodeficiency virus—HIV, hepatitis B virus—HBV, hepatitis C virus—HCV), malignant disease, as well as the participant’s refusal to participate in the research. We also excluded COVID-19 patients that required treatment in the intensive care unit or patients with fatal outcome. 

The assessment of the clinical stage of the severity of the disease at the time of diagnosis and/or hospitalization was performed according to the guidelines of the National Protocol for COVID-19 version 11, which was current at that time. Patients were divided into three groups depending on the severity of disease at the time of admission: 1. asymptomatic/mildly symptomatic, 2. medium severity patients with X-ray signs of pneumonia and 3. severe patients with severe pneumonia, signs of cytokine storm or acute respiratory distress syndrome (ARDS) and signs of multiorgan dysfunction.

An epidemiological questionnaire was used to collect demographic and epidemiological data. To collect the results of clinical and laboratory tests, during the recruitment and monitoring of patients (arterial hypertension, obesity, diabetes), data obtained by a specialist examination of the patient, as well as data from the medical history, were used. The database was created through the server of the Faculty of Medicine of the University of Belgrade (“RedCap system”, available online: http://med.bg.ac.rs/?page_id=20953, accessed on 25 September 2023).

Blood samples for DNA isolation were obtained during the study recruitment phase. DNA isolation was performed on the EDTA-anticoagulated peripheral blood obtained from the study participants using PureLink™ Genomic DNA Mini Kit (ThermoFisher Scientific, Waltham, MA, USA). Gene polymorphisms for regulatory and catalytic proteins involved in the regulation of redox homeostasis (*Nrf2 rs6721961*, *GSTM1*, *GSTT1*, *GSTP1 rs1695*, *GSTO1 rs4925*, *GSTO2 rs156697*, *GSTM3 rs1332018*, *SOD2 rs4880*, *GPX1 rs1050450* and *GPX3 rs8177412*) were determined with previously described PCR-based methods [19,29,31,33]. Levels of non-specific markers of inflammation (interleukin-6, C-reactive protein, fibrinogen, ferritin), complete blood count, biochemical markers of organ damage (urea, creatinine, transaminases and lactate dehydrogenase) and indicators of coagulation status (d- dimer, fibrinogen) were determined using methods within the routine laboratory practice of the University Clinical Center of Serbia. 

Depending on the type of variable and the type of distribution, the description of the data was presented as a total number (n) and percentage, mean +/− standard deviation or median (minimum-maximum value). A 95% confidence interval was used to estimate the population parameters. The methods for testing statistical hypotheses were t-test, χ2 test and analysis of variance. Ordinal logistic regression was used to model the association of dependent variables with potential predictors. During the initial analysis, all p values for both goodness of fit as well as the proportionality of the odds of the models tested were >0.05. Namely, selected laboratory parameters were adjusted to sociodemographic data (age, gender and smoking status) and relevant clinical background (hypertension, diabetes mellitus and obesity), whereas the assessed polymorphisms were additionally adjusted to inflammatory markers (leukocyte, lymphocyte and platelet count, IL-6, CRP, fibrinogen, ferritin and d-dimmer concentration).Values of p less than 0.05 were considered as an indicator of statistical significance. All data was processed in “IBM SPSS Statistics version 22” software (“SPSS Inc., Chicago, IL, USA”) or the software package “R software environment” (“R Core Team (2019)”).

## 5. Conclusions

In conclusion, clinical parameters such as lymphocyte count, CRP, IL6, AST and LDH levels as well as *GPX3* variant allele were associated with the risk of development more severe forms of COVID-19. According to the results of our study, simultaneous determination of non-specific markers of inflammation, complete blood count along with *GPX3* genotype would represent a specific set of markers designed for specific patients (aged, obese, diabetic and hypertensive smokers), exhibiting increased risk for severe forms of COVID-19. These findings could be used as supportive tools helpful in timely identification of such patients with the intention of enabling prevention, proper treatment strategies and hopefully reducing COVID-19 mortality rate in severely or critically ill patients.

## Figures and Tables

**Table 1:** Basic demographic and clinical data of COVID-19 patients.

Parameter	Value
Disease form	n (%)
1	56 (21.1%)
2	141 (53.2%)
3	68 (25.7%)
Age ^a^	56.46 ± 14.70
Gender n (%)	
Female	103 (39%)
Male	162 (61%)
Hypertension n (%)	
No	121 (50%)
Yes	120 (50%)
Diabetes mellitus n (%)	
No	230 (87%)
Yes	35 (13%)
BMI kg/m^2^ n (%)	
>30	83 (32%)
<30	176 (68%)
Smoking habit, n (%)	
No	139 (55%)
Ceased smoking	87 (34%)
Yes	28 (11%)

^a^ The value is presented as mean ± SD; 1. asymptomatic/mildly symptomatic, 2. medium severity patients with X-ray signs of pneumonia and 3. severe patients with severe pneumonia, signs of cytokine storm or acute respiratory distress syndrome and signs of multiorgan dysfunction.

**Table 2:** Selected laboratory parameters of COVID-19 patients at admission.

Parameter	Value ^a^	Referent Range and Units	Number of Patients with Laboratory Parameters Outside of Referent Range n (%) ^b^
Leucocyte count	5.80 (2.00–19.00)	3.4–9.7 × 10^9^/L	↓59 (22%)
Lymphocyte count	1.12 (0.27–9.68)	1.2–3.4 × 10^9^/L	85↓(32%)
Platelet count	201 (62–727)	150–450 × 10^9^/L	58↓ (22%)
IL-6	26 (1.40–205.50)	0–8 pg/L	136↑ (51%)
CRP	48.20 (1.10–355.40)	0–10 mg/L	194↑ (73%)
Ferritin	538.30 (10.10–4937.20)	Female 5–159 Male 28–397 ng/L	85↑ (32%)
Fibrinogen	4.00 (0.42–9.00)	1.8–3.5 g/L	97↑ (37%)
d-dimer	0.52 (0.18–17.05)	<0.5 mg/L	133↑ (50%)
Urea	5.40 (2.0–95)	2.5–7.5 mmol/L	39↑ (15%)
Creatinine	90 (47–539)	59–104 µmol/L	58↑ (22%)
AST	34 (12–152)	0–37 U/L	89↑ (34%)
ALT	43 (11–205)	14–63 U/L	49↑ (19%)
LDH	254 (106–2001)	85–227 U/L	128↑ (48%)

^a^ Median (min–max); IL-6—interleukin 6, CRP—C-reactive protein, AST—aspartate aminotransferase, ALT—alanine aminotransferase, LDH—lactate dehydrogenase; ^b^ ↓—below the lower limit of normal range, ↑—above the upper limit of normal range.

**Table 3:** Estimated risk of developing a more severe form of COVID-19 for selected laboratory parameters.

Parameter	Cut-Offs	OR	95% CI	*P*
Leucocyte count	<3.4 × 10^9^/L	0.47	0.22–1.00	0.050
Lymphocyte count	<1.0 × 10^9^/L	2.97	1.49–5.92	0.002
Platelets	<100 × 10^9^/L	0.91	0.42–1.98	0.813
IL-6	>8 pg/L	8.52	2.48–29.28	0.001
CRP	>10 mg/L	10.97	4.16–28.96	<0.001
Ferritin	>500 ng/L	1.92	0.84–4.36	0.121
Fibrinogen	>3.5 g/L	2.29	1.09–4.81	0.029
d-dimer	>0.5 mg/L	1.37	0.68–2.79	0.382
Urea	>7.5 mmol/L	0.67	0.29–1.58	0.362
Creatinine	>104 µmol/L	1.41	0.66–3.04	0.376
AST	>37 U/L	2.25	1.13–4.49	0.021
ALT	>63 U/L	1.16	0.53–2.54	0.703
LDH	>227 U/L	4.76	2.22–10.22	<0.001

IL-6—interleukin 6, CRP—C-reactive protein, AST—aspartate aminotransferase, ALT—alanine aminotransferase, LDH—lactate dehydrogenase; odds ratio (OR) adjusted to gender, age, comorbidities (hypertension, diabetes mellitus, obesity) and smoking status; CI—confidence interval.

**Table 4:** The association of gene polymorphisms for proteins involved in the redox homeostasis regulation with the risk of severe forms of COVID-19.

Variant vs. Referent Genotypes	OR	95% CI	*P*
Deletion polymorphisms ^a^			
*GSTM1 null* vs. *GSTM1 active*	0.87	0.46–1.66	0.673
*GSTT1 null* vs. *GSTT1 active*	0.75	0.35–1.60	0.455
Single-nucleotide polymorphisms in non-coding regions ^b^			
Nrf2*CA + AA vs. Nrf2*CC	0.80	0.4–1.62	0.538
GSTM3* AC + CC vs. GSTM3*AA	1.76	0.91–3.41	0.091
GPX3*TC + CC vs. GPX3*TT	1.51	0.75–3.03	0.247
Single-nucleotide polymorphisms in coding regions ^c^			
GSTP1*Ile/Val + Val/Val vs. GSTP1*Ile/Ile	1.02	0.35–2.94	0.978
GSTO1* Ala/Asp + Asp/Asp vs. GSTO1*Ala/Ala	0.99	0.53–1.85	0.968
GSTO2**Asn/Asp + AspAsp vs. GSTO2*Asn/Asn	1.23	0.62–2.46	0.551
SOD2*Ala/Val + Val/Val vs. SOD2*Ala/Ala	0.65	0–1.43	0.287
GPX1*Pro/Leu + Leu/Leu vs. GPX1* Pro/Pro	1.02	0.55–1.88	0.952

^a^ no enzyme present; ^b^ affecting enzyme expression; ^c^ affecting enzyme activity; odds ratio (OR) adjusted to gender, age, comorbidities (hypertension, diabetes mellitus, obesity) and smoking status; CI—confidence interval.

**Table 5:** The advanced adjusted analysis of gene polymorphisms for proteins involved in the redox homeostasis regulation of with the risk of developing a more severe form of COVID-19.

Variant vs. Referent Genotypes	OR	95% CI	*P*
Deletion polymorphisms ^a^			
*GSTM1null* vs. *GSTM1 active*	1.01	0.50–2.05	0.982
*GSTT1 null* vs. *GSTT1 active*	1.58	0.65–3.82	0.315
Single-nucleotide polymorphisms in non-coding regions ^b^			
Nrf2*CA + AA vs. Nrf*CC	0.62	0.28–1.40	0.253
GSTM3* AC + CC vs. GSTM3*AA	1.05	0.51–2.18	0.890
GPX3*TC + CC vs. GPX3*TT	2.42	1.08–5.40	0.032
Single-nucleotide polymorphisms in coding regions ^c^			
GSTP1*Ile/Val + Val/Val vs. GSTP1*Ile/Ile	1.94	0.55–6.87	0.305
GSTO1* Ala/Asp + Asp/Asp vs. GSTO1*Ala/Ala	1.33	0.66–2.72	0.427
GSTO2**Asn/Asp + AspAsp vs GSTO2*Asn/Asn	1.05	0.49–2.28	0.892
SOD2*Ala/Val + Val/Val vs. SOD2*Ala/Ala	0.72	0.31–1.70	0.460
GPX1*Pro/Leu + Leu/Leu vs. GPX1* Pro/Pro	1.45	0.71–2.94	0.309

^a^ no enzyme present; ^b^ affecting enzyme expression; ^c^ affecting enzyme activity; odds ratio (OR) adjusted to inflammatory markers (leukocyte, lymphocyte and platelet count, IL-6, CRP, fibrinogen, ferritin, d-dimmer concentration); CI—confidence interval.

## Data Availability

The data supporting reported results are available at the RedCap platform (Research Electronic Data Capture, Vanderbilt University) of Faculty of Medicine University in Belgrade and will be made available by the corresponding authors upon request without undue reservation.
